# Supplementary material for: Asynchronous magnetic resonance elastography: Shear wave speed reconstruction using noise correlation of incoherent waves
Source: Magn Reson Med. 2022 Oct 27;89(3):990–1001. doi: 10.1002/mrm.29502 (PMC9792433; doi:10.1002/mrm.29502)
Supplement: Supplementary file 1 — DATA S1 MATLAB code used to generate the numerical simulations of Figure 1 [file MRM-89-990-s002.zip › k-Wave/helpfiles/kWaveDiffusion.html]

kWaveDiffusion :: Functions (k-Wave)


# kWaveDiffusion

Time-domain simulation of heat diffusion and perfusion.

## Syntax

```
kdiff = kWaveDiffusion(kgrid, medium, source)
kdiff = kWaveDiffusion(kgrid, medium, source, sensor)
kdiff = kWaveDiffusion(kgrid, medium, source, sensor, ...)
kdiff = kWaveDiffusion(kgrid, medium, source, [], ...)
```

## Description

`kWaveDiffusion` is a class definition for the time-domain solution of the diffusion equation or Pennes' bioheat equation in 1D, 2D, and 3D. In addition to heat diffusion, Pennes' bioheat equation accounts for advective heat loss due to tissue perfusion (the flow of blood through tissue), and heat deposition (e.g., due to ultrasound absorption) [1]. The computation is based on a k-space pseudospectral scheme in which spatial gradients are calculated using the Fourier collocation spectral method, and temporal gradients are calculated using a k-space corrected finite difference scheme. For a homogeneous medium, the formulation is exact and unconditionally stable. For a heterogeneous medium, the time scheme allows larger time-steps to be taken for the same level of accuracy compared to conventional pseudospectral time-domain methods.

The most general partial differential equation solved by the `kWaveDiffusion` class is given by

```
A * dT/dt = div(Kt * grad(T)) - B * (T - Ta) + Q
```

where

```
A  = density [kg/m^3] * specific heat capacity [J/(kg.K)]
Kt = thermal conductivity [W/(m.K)]
B  = blood density [kg/m^3] * blood specific heat [J/(kg.K)] * blood perfusion rate [1/s]    
Ta = arterial temperature (blood ambient temperature) [degC]
Q  = volume rate of heat deposition [W/m^3]
```

In a homogeneous medium, the thermal coefficients are related to the diffusion coefficient (or thermal diffusivity) by

```
diffusion coefficient [m^2/s] = Kt / A
```

Note, when the diffusion coefficient is specified instead of the individual thermal coefficients, the equation that is solved is

```
dT/dt = div(D * grad(T))
```

For non-constant coefficients, this differs from the conventional heat equation (where the diffusion coefficient is taken outside the divergence operator). For convenience, the thermal coefficients related to perfusion can also be combined to give a single "perfusion coefficient" given by

```
perfusion coefficient [1/s] = B / A
```

The input parameters are assigned as fields to four input structures (`kgrid`, `medium`, `source`, and `sensor`) in the same way as the other models in the k-Wave toolbox. These structures define the properties of the computational grid, the distribution of medium properties, source terms, and the locations of the sensor points used to record the evolution of the temperature field over time.

The medium parameters can each be specified as a single scalar values in SI units (for homogeneous coefficients), or as matrices the same size as the computational grid (for heterogeneous coefficients).

The initial temperature distribution is specified by assigning a single scalar value or a matrix (the same size as the computational grid) to `source.T0`. A heat source can also be specified in the same way by defining `source.Q` (the volume rate of heat deposition).

The time history of the temperature field can be recorded automatically by specifying a series of sensor locations using sensor.mask. This is defined as a binary matrix (i.e., a matrix of 1's and 0's with the same dimensions as the computational grid) representing the grid points within the computational grid that will record the temperature field at each time step. The current sensor data can be queried at any point using the property `kdiff.sensor_data` (where `kdiff` is the name of the `kWaveDiffusion` object). The `sensor_data` is returned using MATLAB's standard column-wise linear matrix index ordering, indexed as `sensor_data(sensor_point_index, time_index)`. If no time dependent output is required, the sensor input can be replaced with an empty array `[]`.

After an object of the `kWaveDiffusion` class is created, the simulation is run by calling the method `kdiff.takeTimeStep(Nt, dt)`, where `kdiff` is the object name, `Nt` is the number of time steps to take, and `dt` is the size of the time step. During the simulation, a visualisation of the temperature field is displayed. The current temperature can be queried (or modified) at any point using the property `kdiff.T`.

[1] Pennes, H. H. (1948). Analysis of tissue and arterial blood temperatures in the resting human forearm. Journal of Applied Physiology, 1(2), 93-122.

## Inputs

|  |  |
| --- | --- |
| `kgrid` | grid object returned by `kWaveGrid` |
|  |  |
| `medium.diffusion_coeff` | diffusion coefficient [m^2/s] |
|  | OR |
| `medium.density` | tissue mass density [kg/m^3] |
| `medium.specific_heat` | tissue specific heat capacity [J/(kg.K)] |
| `medium.thermal_conductivity` | tissue thermal conductivity [W/(m.K)] |
|  |  |
| `medium.perfusion_coeff` | perfusion coefficient [1/s] |
|  | OR |
| `medium.blood_density` | blood mass density [kg/m^3] |
| `medium.blood_specific_heat` | blood specific heat capacity [J/(kg.K)] |
| `medium.blood_perfusion_rate` | blood perfusion rate [1/s] (volumetric flow rate per unit volume) |
|  |  |
| `medium.blood_ambient_temperature` | ambient blood temperature within perfused tissue regions [degC] |
|  |  |
| `medium.diffusion_coeff_ref` | reference diffusion coefficient used within the k-space operator (default = 'max') |
| `medium.perfusion_coeff_ref` | reference perfusion coefficient used within the k-space operator (default = 'max') |
|  |  |
| `source.T0` | initial temperature distribution [degC] |
| `source.Q` | volume rate of heat deposition [W/m^3] (note, `medium.density` and `medium.specific_heat` must be defined when using `source.Q`) |
|  |  |
| `sensor.mask` | binary grid specifying where the temperature is recorded at each time step |

## Optional Inputs

Optional 'string', value pairs that may be used to modify the default computational settings.

| Input | Valid Settings | Default | Description |
| --- | --- | --- | --- |
| `'DisplayUpdates'` | *(Boolean scalar)* | `true` | Boolean controlling whether details of the simulation are printed to the MATLAB command line. |
| `'MovieArgs'` | *(string cell array)* | `{}` | Settings for `VideoWriter`. Parameters must be given as {'param', value, ...} pairs within a cell array (default = {}), where 'param' corresponds to a writable property of a VideoWriter object. |
| `'MovieName'` | *(string)* | `'date-time-kWaveDiffusion'` | Name of the movie produced when `'RecordMovie'` is set to `true`. |
| `'MovieProfile'` | *(string)* | `'Uncompressed AVI'` | Profile input passed to `VideoWriter`. |
| `'PlotFreq'` | *(integer numeric scalar)* | `10` | The number of iterations which must pass before the simulation plot is updated. |
| `'PlotScale'` | *(numeric two element vector)* or `'auto'` | `'auto'` | [min, max] values used to control the scaling for `imagesc` (visualisation). If set to `'auto'`, a symmetric plot scale is chosen automatically for each plot frame. |
| `'PlotSim'` | *(Boolean scalar)* | `true` | Boolean controlling whether the simulation iterations are progressively plotted. |
| `'RecordMovie'` | *(Boolean scalar)* | `false` | Boolean controlling whether the displayed image frames are captured and stored as a movie using `VideoWriter`. |

## Outputs

|  |  |
| --- | --- |
| `kdiff` | `kWaveDiffusion` object which can be used to run thermal simulations using the diffusion equation or Pennes bioheat equation |

## Dynamic Properties Properties which can be queried or modified after the object is created. | Fieldname | Description | | --- | --- | | `cem43` | thermal dose given in cumulative equivalent minutes (cem) relative to T = 43 degC [mins] | | `T` | current temperature field [degC] | | `Q` | volume rate of heat deposition [W/m^3] | Static Properties Properties which can be queried, but not modified, after the object is created. | Fieldname | Description | | --- | --- | | `dt_limit` | maximum time step for which the simulation remains stable [s] | | `lesion_map` | binary matrix of `cem43 >= 240` mins | | `lesion_size` | total size of `lesion_map` (distance in 1D [m], area in 2D [m^2], volume in 3D [m^3]) | | `sensor_data` | time varying temperature recorded at the sensor positions given by `sensor.mask` [degC] | Methods | Methodname | Description | | --- | --- | | `plotTemp` | plot current temperature field in current figure window | | `setOptionalInputs('string', value, ...)` | modify the optional inputs after the object is created | | `takeTimeStep(Nt, dt)` | calculate the given number of time steps of the temperature field | See Also `bioheatExact`
